# Adverse reactions of immune checkpoint inhibitors combined with acetaminophen/non-steroidal anti-inflammatory drugs (NSAIDs): a pharmacovigilance analysis of drug–drug interactions

**DOI:** 10.3389/fphar.2026.1833275

**Published:** 2026-10-09

**Authors:** Xiayang Ren, Lihui Xue, Yanfeng Wang, Jian Xiao

**Affiliations:** 1 Department of Pharmacy, National Cancer Center, National Clinical Research Center for Cancer, Cancer Hospital, Chinese Academy of Medical Sciences and Peking Union Medical College, Beijing, China; 2 Department of Pharmacy, Beijing Chaoyang District Sanhuan Cancer Hospital, Beijing, China; 3 Department of Comprehensive Oncology, National Cancer Center, National Clinical Research Center for Cancer, Cancer Hospital, Chinese Academy of Medical Sciences and Peking Union Medical College, Beijing, China; 4 Department of Pharmacy, Xiangya Hospital, Central South University, Changsha, China

**Keywords:** acetaminophen, drug–drug interaction, FDA adverse event reporting system (FAERS), immune checkpoint inhibitors, non-steroidal anti-inflammatory drugs, pharmacovigilance

## Abstract

**Background:**

The combination of immune checkpoint inhibitors (ICIs) with acetaminophen (APAP) and non-steroidal anti-inflammatory drugs (NSAIDs) is commonly employed in cancer treatment. However, potential drug–drug interactions (DDIs) between these agents remain poorly understood. Our study aimed to identify interaction signals associated with the concomitant use of ICIs and APAP/NSAIDs.

**Methods:**

Data were obtained from the US FDA Adverse Event Reporting System (FAERS) covering the period from 1 January 2014, to 30 June 2025. Disproportionality analysis was conducted for data mining by calculating the reporting odds ratio and 95% confidence interval (95% CI). Adjusted RORs were analyzed using logistic regression, accounting for age, sex, and reporting year. Further confirmation was performed using both additive and multiplicative models.

**Results:**

We identified 4,613 reports related to the combination of immune checkpoint inhibitors (ICIs) with acetaminophen (APAP) and non-steroidal anti-inflammatory drugs (NSAIDs). Significant interaction signals were observed for gastrointestinal disorders (adjusted ROR: 1.57, 95% CI: 1.41–1.70, additive model: 0.0549), investigations (adjusted ROR: 1.27, 95% CI: 1.13–1.43, additive model: 0.3847, multiplicative model: 1.3135), renal and urinary disorders (adjusted ROR: 4.68, 95% CI: 4.19–5.24, additive model: 3.5771, multiplicative model: 3.6312), and skin and subcutaneous tissue disorders (adjusted ROR: 1.64, 95% CI: 1.47–1.83, additive model: 0.6818, multiplicative model: 1.5996). Subset analysis revealed positive interaction signals for the combination of ICIs with APAP in gastrointestinal disorders and investigations, for all APAP/NSAIDs combinations with ICIs in renal and urinary disorders, and for the combination of NSAIDs with ICIs in skin and subcutaneous tissue disorders.

**Conclusion:**

Based on data from the FAERS, four systemic disorders were identified to be associated with drug–drug interactions (DDIs) resulting from the combined use of ICIs and APAP/NSAIDs. Further preclinical trials and well-designed, large-scale clinical studies are needed to explore the potential pharmacodynamic crosstalk, alterations in inflammatory pathway and changes in the tumor immune microenvironment induced by the co-administration of ICIs with APAP/NSAIDs. This will help deepen the understanding of DDIs associated with this combination therapy.

## 1 Introduction

The advent of immune checkpoint inhibitors (ICIs), including antibodies targeting cytotoxic T-lymphocyte-associated protein 4 (CTLA-4), programmed cell death protein 1 (PD-1), and its ligand (PD-L1), has fundamentally transformed the therapeutic landscape for a broad spectrum of malignancies (Michielin et al., 2022). By reinvigorating exhausted T cells and overcoming tumor-mediated immune suppression, ICIs have exhibited unprecedented and durable clinical responses across diverse cancer types, facilitating their rapid integration into standard-of-care regimens—both as monotherapy and in combination with other treatment modalities (Ribas and Wolchok, 2018). This widespread andoften prolonged use underscores the critical need for a comprehensive understanding of their safety profile and potential interactions with concomitant medications.

Concurrently, acetaminophen (APAP) and non-steroidal anti-inflammatory drugs (NSAIDs) remain among the most frequently prescribed medications globally for the management of cancer-related pain, inflammation, and fever. Their analgesic and anti-inflammatory effects, primarily mediated through the inhibition of cyclooxygenase (COX) enzymes, render them a cornerstone of supportive care in oncology (Khalil et al., 2024). As a result, the co-administration of APAP/NSAIDs with ICI therapy is a commonplace clinical scenario, fueled by the need to alleviate symptoms and enhance patients’ quality of life during cancer treatment (Sohi et al., 2021).

However, this frequent co-administration raises important pharmacological concerns. Both ICIs and APAP/NSAIDs profoundly impact the immune system, albeit through distinct mechanisms. ICIs induce a systemic, pro-inflammatory state essential for antitumor immunity but also predispose patients to immune-related adverse events (irAEs). In contrast, the cyclooxygenase pathways, inhibited by NSAIDs, have been implicated in promoting tumor growth, progression, and immunosuppression (Zelenay et al., 2015; Bonavita et al., 2020). The potential pharmacokinetic and, more critically, pharmacodynamic interactions between these two drug classes could theoretically alter the efficacy or toxicity profiles of either agent (Sharma and Allison, 2020).

The concomitant use of multiple drugs can lead to drug–drug interactions (DDIs), which can alter the efficacy and safety profiles of medications, and may consequently result in toxicities and treatment failure (Blower et al., 2005). However, there is a paucity of research investigating DDIs focused specifically on the co-administration of ICIs and APAP/NSAIDs. Existing research on the co-administration of ICIs and APAP/NSAIDs has primarily focused on the clinical outcomes, yielding inconsistent and complex findings (Stone et al., 2026; Zhang et al., 2021; Mao et al., 2022; Li et al., 2026). However, research addressing their safety remains limited, with only three relevant studies reported to date. One study, which performed a multivariate regression analysis from the FAERS database, found that aspirin use was associated with an increased risk of irAEs across a pan-cancer setting (Yang et al., 2023). Notably, low-dose aspirin is currently primarily used for cardiovascular protection. Unlike other NSAIDs, it is not clinically employed for antipyretic or analgesic purposes. Additionally, two studies have confirmed an association between kidney injury and NSAID use in the context of ICI therapy. One meta-analysis investigating the risk factors of acute kidney injury (AKI) associated with ICIs identified NSAID use as one of the significant contributing factors (Liu et al., 2023). Another pharmacovigilance study, which analyzed the World Health Organization (WHO) international pharmacovigilance database Vigibase, demonstrated an increased reporting of nephritis in patients receiving both NSAIDs and ICIs (Laurent et al., 2025). However, neither of these studies employed methodologies specifically designed to evaluate DDIs. In general, the aforementioned studies only described simple correlation phenomena without rigorous verification of interaction effects, resulting in a weak clinical base for the safe concurrent medication of ICIs and anti-inflammatory analgesics. Currently, large-scale, standardized evidence regarding DDIs involving the co-administration of ICIs and APAP/NSAIDs remains insufficient and has not been comprehensively summarized in the existing literature. Against this background, our study further supplements real-world pharmacovigilance evidence in this field and strengthens the research positioning in the existing ICI-DDI evidence system.

To better understand the potential DDIs between ICIs and APAP/NSAIDs, it is essential to gather real-world data. We conducted descriptive and disproportionality analyses using the United States Food and Drug Administration (FDA) Adverse Event Reporting System (FAERS) database to investigate the potential harmful effects of these co-administrations. Based on real-world spontaneous reporting big data, our study characterized disproportional signals of ICI-associated analgesic interactions, described the clinical features and distribution patterns of adverse events, and provided preliminary reference evidence for rational clinical medication and risk awareness.

## 2 Methods

### 2.1 Data source

The FAERS database was used for this retrospective pharmacovigilance study (U.S. Food and Drug Administration, 2021). The raw data can be obtained from the FAERS database at the following link: FAERS Quarterly Data Extract Files (fda.gov). The FAERS database includes adverse event (AE) reports from healthcare professionals, patients, and manufacturers worldwide, and the data are publicly accessible. Structurally, FAERS consists of seven standardized core data tables, including patient demographic information (DEMO), drug administration records (DRUG), adverse event terms (REAC), patient outcomes (OUTC), report sources (RPSR), therapy duration information (THER) and indicate information (INDI), which enables integrated retrieval of medication exposure, combined medication information and corresponding adverse reactions. To eliminate duplicate reports, the most recent FDA_DT was selected when the CASEID was identical. If both CASEID and FDA_DT were identical, the PRIMARY_ID with the higher value was selected, following the FAERS user instructions (U.S. Food and Drug Administration, 2021). Data were extracted for the period from the first quarter (Q1) of 2014 to the second quarter (Q2) of 2025.

### 2.2 Procedures

Data were obtained from the REAC files according to the Medical Dictionary for Regulatory Activities (MedDRA, version 23.0) at the preferred term (PT) level, based on all 27 Standardized MedDRA Query System Organ Class (SOC) (Maintenance and support service organization). The 27 SOCs are listed in Supplementary Table S1. The DRUG file contained records of generic drug names, with the role_code selected as either ‘PS’ (primary suspect) or ‘SS’ (secondary suspect). Combined immunotherapy drugs included anti-PD-1 agents (pembrolizumab, nivolumab, cemiplimab, dostarlimab, prolongolimab, tislelizumab, toripalimab, sintilimab, camrelizumab, penpulimab, zimberelimab, and serplulimab), anti-PD-L1 agents (atezolizumab, durvalumab, avelumab, envafolimab, and sugemalimab), and anti-CTLA4 agents (ipilimumab and tremelimumab). APAP/NSAIDs included acetaminophen, non-selective COX inhibitors, and selective COX-2 inhibitors, comprising a total of 33 medications (Supplementary Table S2). The inclusion criteria were that the role_code (PS) included both ICIs and either APAP or NSAIDs, with clear records of adverse events. The exclusion criteria were reports with incomplete key information and duplicate reports. Of note, three-step data cleaning was conducted before analysis (Figure 1). First, duplicates and multiple cases or reports were removed, and only the most recent version of each report without duplication was extracted. Second, a drug event combination of ICIs and APAP/NSAIDs was established, and only those AE reports with reported roles of drugs as “suspect” were included. Finally, based on different therapeutic regimens, total reports were divided into four subgroups: (1). No ICIs, no APAP/NSAIDS, (2). APAP/NSAIDS only, no ICIs, (3). ICIs only, no APAP/NSAIDS, (4). ICIs combined with APAP/NSAIDS. Descriptive analyses were conducted to synthesize the clinical features of patients receiving ICIs in combination with APAP/NSAIDs using data from the FAERS database.

**FIGURE 1:**
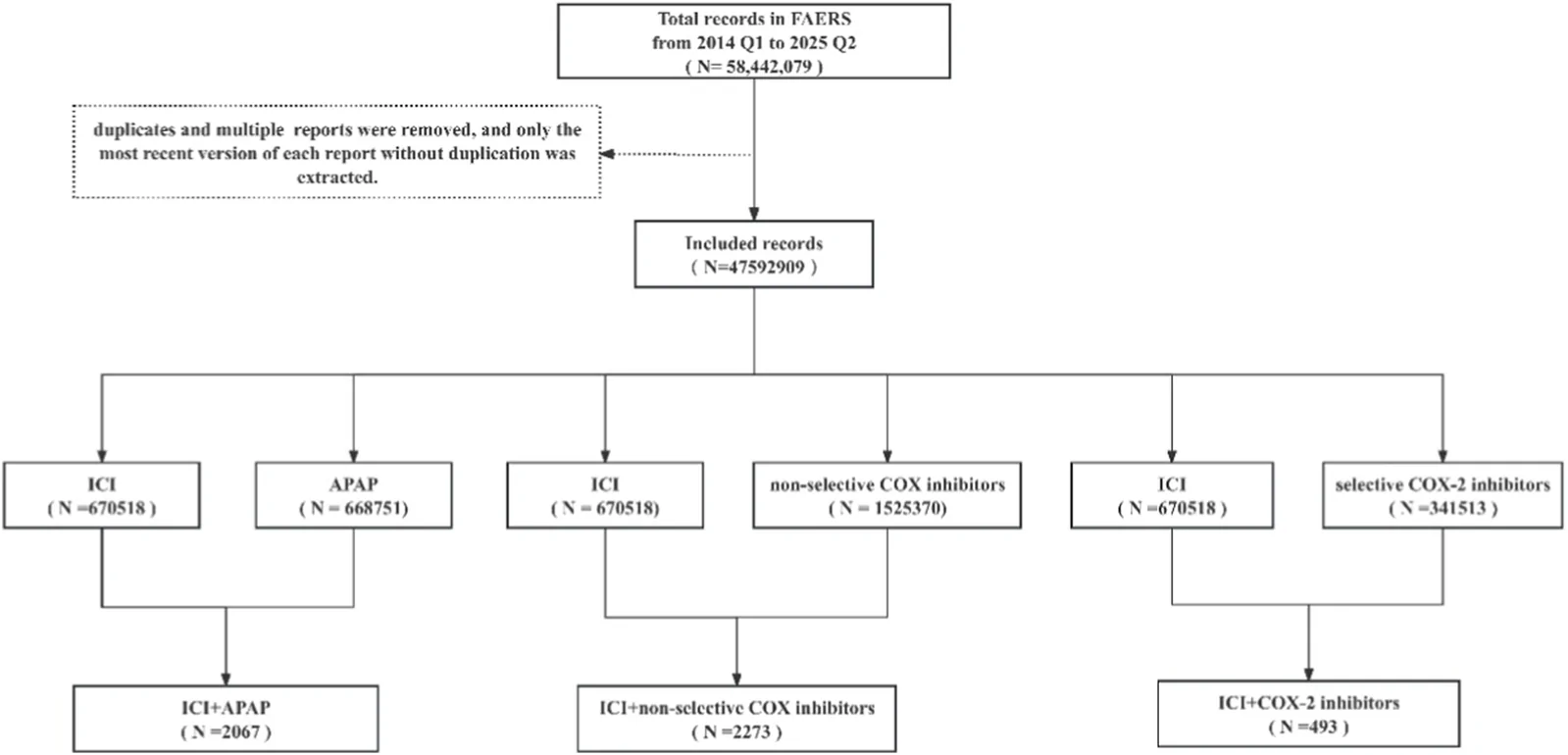
Flowchart for data processing. FAERS, US Food and Drug Administration (FDA) Adverse Event Reporting System; ICI, immune checkpoint inhibitors; Q, quarter; APAP, acetaminophen; COX, cyclooxygenase.

### 2.3 Data analysis

In spontaneous reporting databases, the detection of potential drug interactions relies on the observation that a suspected adverse event (AE) occurs more frequently when two drugs are used in combination than when they are used individually (van Puijenbroek et al., 2000; Yue et al., 2014; Capogrosso Sansone et al., 2017). To analyze this phenomenon, the reports were categorized into three index groups: (i) patients exposed to ICIs alone, without APAP or NSAIDs; (ii) patients exposed to APAP/NSAIDs alone, without ICIs; and (iii) patients exposed to both ICIs and APAP/NSAIDs at the time of the event. The reference group comprised patients who were not exposed to either ICIs or APAP/NSAIDs.

Disproportionality was assessed using the reporting odds ratio (ROR), along with the corresponding 95% confidence interval (95% CI). A result was considered statistically significant when the lower bound of the 95% CI exceeded 1, provided that at least three cases of interest were reported (van Puijenbroek et al., 2002). To control for potential confounders, crude RORs were recalculated using unconditional logistic regression, adjusting for age, sex, and reporting year (Noguchi et al., 2019). The logistic regression model used was as follows: Log(risk of the event) = *β*0 + *β*1 (ICIs) + *β*2 (APAP/NSAIDs) + *β*3 (ICIs × APAP/NSAIDs) + *β*4 (age) + *β*5 (gender) + *β*6 (reporting year). An interaction was considered significant if the combination of drugs was associated with an enhanced ROR compared to the other index groups (Thakrar et al., 2007).

Various methods are available for detecting DDI signals. The logistic regression model is a statistical method used to identify DDI signals from a spontaneous reporting system. The ROR, similar to the odds ratio, is a statistical model that can be adjusted for age and gender using logistic regression. The additive and multiplicative models are also used to detect potential DDI signals. In the additive model, drug-related risks increase additively, whereas in the multiplicative model, they increase synergistically (Noguchi et al., 2019). Given that no universally accepted statistical method exists for detecting DDI signals from spontaneous reporting data, we employed the adjusted ROR and additive/multiplicative models for signal detection.

To evaluate the consistency and reliability of the detected DDIs, both the multiplicative and additive models were used (Noguchi et al., 2019). The analysis provided a threshold for detecting DDI signals. In the multiplicative model, the risk associated with a drug is multiplied by the background risk, while in the additive model, the risk associated with the drug is added to the background risk. In the multiplicative model, a positive interaction is indicated when: The risk (drug 1 × drug 2)/((risk (drug 1) × risk (drug 2)) >1. In the additive model, a positive interaction is indicated when: risk (drug 1 × drug 2) – (risk (drug 1) + risk (drug 2)) >0. If the interaction term exceeds 0 in the additive model or one in the multiplicative model, it suggests a positive drug interaction (Noguchi et al., 2020). Data management and analyses were conducted using SPSS (version 22.0; IBM Corp., Armonk, NY, United States of America).

## 3 Results

### 3.1 Descriptive analysis

From January 2014 to June 2025, a total of 58, 442, 079 reports were extracted from the FAERS database, of which 47, 592, 909 reports were included in the final analysis. Among these, 4,613 reports involved the co-administration of ICIs and APAP/NSAIDs. The clinical characteristics of the patients are summarized in Table 1. The median age of patients was 64 years (IQR: 53–72 years). The proportion of male patients (47.56%) was slightly higher than that of female patients (44.00%). Healthcare professionals submitted a significant proportion of the reports (43.67%). Europe accounted for the highest rate of adverse drug reactions (ADRs) (49.00%), followed by Asia (23.78%), North America (23.00%), Oceania (1.44%), and Africa (0.11%). The number of reports steadily increased over time, from 0.56% in 2014 to 19.56% in 2024.

**TABLE 1:** Characteristics of patients treated with immune checkpoint inhibitors combined with acetaminophen/non-steroidal anti-inflammatory drugs (NSAIDs) in the FAERS database (1 January 2014, to 30 June 2025).

Characteristics	Overall
Total number of cases	900
Patient’s age, years, median (Q1–Q3)	
<18 years	26 (2.89%)
18–65 years	336 (37.33%)
≥65 years	365 (40.56%)
Unknown	173 (19.22%)
Patient’s gender [n (%)]	​
Male	428 (47.56%)
Female	396 (44.00%)
Unknown	76 (8.44%)
Type of reporter	​
Health professional	393 (43.67%)
Non-health professional	70 (7.78%)
Unknown	437 (48.56%)
Outcome	​
Hospitalization	402 (44.67%)
Life-threatening	51 (5.67%)
Death	142 (15.78%)
Reported regions	​
North America	207 (23.00%)
South America	0 (0.00%)
Europe	441 (49.00%)
Asia	214 (23.78%)
Oceania	13 (1.44%)
Africa	1 (0.11%)
Unknown	24 (2.67%)
Reported year	​
2014	5 (0.56%)
2015	5 (0.56%)
2016	8 (0.89%)
2017	37 (4.11%)
2018	68 (7.56%)
2019	75 (8.33%)
2020	78 (8.67%)
2021	80 (8.89%)
2022	132 (14.67%)
2023	106 (11.78%)
2024	176 (19.56%)
2025	130 (14.44%)

### 3.2 Disproportionality analysis

The signal detection results for DDIs associated with theco-administration of ICIs and APAP/NSAIDs across all 27 SOCs are presented in Table 2. The crude and adjusted RORs and 95% CIs for all comparisons are provided using a disproportionality method. DDIs were estimated using both additive and multiplicative models. Positive signals were identified in four SOCs: gastrointestinal disorders, investigations, renal and urinary disorders, and skin and subcutaneous tissue disorders (Table 3). For gastrointestinal disorders, the adjusted ROR for APAP/NSAIDs alone was 1.28 (95% CI: 1.28–1.29), and for ICIs alone, it was 1.24 (95% CI: 1.23–1.25). A signal increase was observed with the concomitant use of ICIs and APAP/NSAIDs (adjusted ROR: 1.57, 95% CI: 1.41–1.70). This positive interaction signal was further supported by the additive model (0.0549). For investigations, the adjusted ROR for APAP/NSAIDs alone was 1.05 (95% CI: 1.04–1.06), and for ICIs alone, it was 1.07 (95% CI: 1.06–1.08). An increase in the signal was detected with the concomitant use of ICIs and APAP/NSAIDs (adjusted ROR: 1.27, 95% CI: 1.13–1.43). The positive interaction signal was supported by both the additive (0.3847) and multiplicative (1.3135) models. For renal and urinary disorders, the adjusted ROR for APAP/NSAIDs alone was 1.07 (95% CI: 1.06–1.09), and for ICIs alone, it was 1.50 (95% CI: 1.48–1.53). The signal for concomitant use of ICIs and APAP/NSAIDs showed a notable increase (adjusted ROR: 4.68, 95% CI: 4.19–5.24). The positive interaction was supported by both the additive (3.5771) and multiplicative (3.6312) models. For skin and subcutaneous tissue disorders, the adjusted ROR for APAP/NSAIDs alone was 0.99 (95% CI: 0.99–1.00), and for ICIs alone, it was 1.02 (95% CI: 1.01–1.04). An increase in the signal was observed for the concomitant use of ICIs and APAP/NSAIDs (adjusted ROR: 1.64, 95% CI: 1.47–1.83). This positive interaction was supported by both the additive (0.6818) and multiplicative (1.5996) models.

**TABLE 2:** Reporting odds ratios (RORs) and drug interaction approaches for immune checkpoint inhibitors (ICIs) and acetaminophen/non-steroidal anti-inflammatory drugs (NSAIDs).

SOC	Exposure	Cases	Non-cases	RORcrude (95%Cl)	RORadj (95%Cl)	Additive model	Multiplicative model
Blood and lymphatic system disorders	No ICIs, no APAP/NSAIDS	725,229	46,867,686	Reference	Reference	−0.6303	1.0959
	APAP/NSAIDS, no ICIs	17,389	1,900,229	0.56 (0.55–0.57)	0.59 (0.58–0.60)		
	ICIs, no APAP/NSAIDS	30,081	640,437	2.76 (2.73–2.80)	2.13 (2.10–2.16)		
	ICIs, APAP/NSAIDS	127	4,486	1.69 (1.42–2.02)	1.32 (1.09–1.60)		
Cardiac disorders	No ICIs, no APAP/NSAIDS	1,007,038	46,585,873	Reference	Reference	−0.6793	0.3935
	APAP/NSAIDS,no ICIs	33,852	1,883,766	0.79 (0.78–0.80)	0.89 (0.88–0.90)		
	ICIs, no APAP/NSAIDS	19,249	651,269	1.29 (1.27–1.31)	1.11 (1.09–1.13)		
	ICIs, APAP/NSAIDS	41	4,572	0.40 (0.30–0.55)	0.27 (0.19–0.40)		
Congenital, familial, and genetic disorders	No ICIs, no APAP/NSAIDS	120,828	47,472,091	Reference	Reference	0.1839	1.2394
	APAP/NSAIDS, no ICIs	4,149	1,913,469	0.82 (0.80–0.85)	0.63 (0.60–0.67)		
	ICIs, no APAP/NSAIDS	480	670,038	0.27 (0.25–0.30)	0.62 (0.56–0.68)		
	ICIs, APAP/NSAIDS	3	4,610	0.28 (0.09–0.86)	0.40 (0.10–1.61)		
Ear and labyrinth disorders	No ICIs, no APAP/NSAIDS	192,359	47,400,574	Reference	Reference	0.8816	3.0390
	APAP/NSAIDS, no ICIs	6,707	1,910,911	0.83 (0.81–0.85)	0.82 (0.80–0.85)		
	ICIs, no APAP/NSAIDS	1,319	669,199	0.47 (0.44–0.49)	0.45 (0.42–0.48)		
	ICIs, APAP/NSAIDS	23	4,590	1.18 (0.78–1.78)	1.27 (0.83–1.95)		
Endocrine disorders	No ICIs, no APAP/NSAIDS	92,454	47,500,458	Reference	Reference	−5.7767	1.1036
	APAP/NSAIDS, no ICIs	2,327	1,915,291	0.49 (0.47–0.51)	0.44 (0.42–0.46)		
	ICIs, no APAP/NSAIDS	22,745	647,773	13.72 (13.52–13.93)	18.34 (18.02–18.67)		
	ICIs, APAP/NSAIDS	88	4,525	7.44 (6.02–9.18)	6.32 (5.01–7.96)		
Eye disorders	No ICIs, no APAP/NSAIDS	903,279	46,689,639	Reference	Reference	0.1880	1.1209
	APAP/NSAIDS, no ICIs	25,736	1,891,882	0.68 (0.67–0.69)	0.67 (0.66–0.68)		
	ICIs, no APAP/NSAIDS	7,278	663,240	0.55 (0.54–0.56)	0.55 (0.53–0.56)		
	ICIs, APAP/NSAIDS	38	4,575	0.42 (0.31–0.58)	0.47 (0.34–0.65)		
Gastrointestinal disorders	No ICIs, no APAP/NSAIDS	3,643,112	43,949,805	Reference	Reference	**0.0549**	0.9966
	APAP/NSAIDS, no ICIs	193,223	1,724,395	1.23 (1.22–1.23)	**1.28 (1.28–1.29)**		
	ICIs, no APAP/NSAIDS	69,590	600,928	1.26 (1.25–1.27)	**1.24 (1.23–1.25)**		
	ICIs, APAP/NSAIDS	586	4,027	1.55 (1.42–1.69)	**1.57 (1.41–1.70)**		
General disorders and administration site conditions	No ICIs, no APAP/NSAIDS	7,920,613	39,672,297	Reference	Reference	−0.2404	0.7420
	APAP/NSAIDS, no ICIs	364,467	1,553,151	1.08 (1.08–1.08)	1.01 (1.00–1.01)		
	ICIs, no APAP/NSAIDS	95,503	575,015	0.81 (0.80–0.81)	0.72 (0.72–0.73)		
	ICIs, APAP/NSAIDS	526	4,087	0.65 (0.59–0.71)	0.63 (0.57–0.69)		
Hepatobiliary disorders	No ICIs, no APAP/NSAIDS	342,020	47,250,894	Reference	Reference	−0.8284	0.5420
	APAP/NSAIDS, no ICIs	29,707	1,887,911	1.86 (1.83–1.88)	1.81 (1.78–1.84)		
	ICIs, no APAP/NSAIDS	25,169	645,349	4.50 (4.44–4.56)	4.04 (3.98–4.10)		
	ICIs, APAP/NSAIDS	176	4,437	4.53 (3.89–5.26)	3.35 (2.82–3.98)		
Immune system disorders	No ICIs, no APAP/NSAIDS	495,653	47,097,279	Reference	Reference	−1.1850	0.4653
	APAP/NSAIDS, no ICIs	49,211	1,868,407	2.21 (2.19–2.23)	2.15 (2.12–2.17)		
	ICIs, no APAP/NSAIDS	7,206	663,312	0.93 (0.90–0.95)	0.87 (0.85–0.90)		
	ICIs, APAP/NSAIDS	51	4,562	0.95 (0.72–1.26)	0.83 (0.61–1.14)		
Infections and infestations	No ICIs, no APAP/NSAIDS	2,427,651	45,165,278	Reference	Reference	−0.1467	0.8359
	APAP/NSAIDS, no ICIs	81,008	1,836,610	0.79 (0.78–0.80)	0.72 (0.72–0.73)		
	ICIs, no APAP/NSAIDS	37,694	632,824	1.05 (1.04–1.06)	0.95 (0.94–0.96)		
	ICIs, APAP/NSAIDS	171	4,442	0.69 (0.60–0.81)	0.60 (0.51–0.71)		
Injury, poisoning, and procedural complications	No ICIs, no APAP/NSAIDS	4,749,571	42,843,350	Reference	Reference	−0.4042	0.5947
	APAP/NSAIDS, no ICIs	254,777	1,662,841	1.25 (1.25–1.26)	1.07 (1.07–1.08)		
	ICIs, no APAP/NSAIDS	42,191	628,327	0.59 (0.59–0.60)	0.45 (0.44–0.45)		
	ICIs, APAP/NSAIDS	216	4,397	0.44 (0.39–0.51)	0.36 (0.31–0.42)		
Investigations	No ICIs, no APAP/NSAIDS	2,562,124	45,030,785	Reference	Reference	**0.3847**	**1.3135**
	APAP/NSAIDS, no ICIs	109,604	1,808,014	1.00 (0.99–1.01)	**1.05 (1.04–1.06)**		
	ICIs, no APAP/NSAIDS	46,980	623,538	1.23 (1.21–1.24)	**1.07 (1.06–1.08)**		
	ICIs, APAP/NSAIDS	425	4,188	1.61 (1.46–1.78)	**1.27 (1.13–1.43)**		
Metabolism and nutrition disorders	No ICIs, no APAP/NSAIDS	906,124	46,686,800	Reference	Reference	−0.2026	0.9704
	APAP/NSAIDS, no ICIs	32,904	1,884,714	0.85 (0.84–0.85)	0.93 (0.91–0.94)		
	ICIs, no APAP/NSAIDS	27,060	643,458	1.99 (1.96–2.01)	1.72 (1.70–1.75)		
	ICIs, APAP/NSAIDS	153	4,460	1.63 (1.39–1.92)	1.34 (1.12–1.61)		
Musculoskeletal and connective tissue disorders	No ICIs, no APAP/NSAIDS	2,235,226	45,357,701	Reference	Reference	−0.7045	0.6928
	APAP/NSAIDS, no ICIs	193,112	1,724,506	1.95 (1.94–1.96)	2.07 (2.06–2.08)		
	ICIs, no APAP/NSAIDS	24,379	646,139	0.71 (0.70–0.71)	0.66 (0.65–0.67)		
	ICIs, APAP/NSAIDS	227	4,386	0.95 (0.84–1.09)	0.85 (0.73–0.98)		
Neoplasms benign, malignant, and unspecified (incl cysts and polyps)	No ICIs, no APAP/NSAIDS	1,251,387	46,341,547	Reference	Reference	−0.1514	2.9540
	APAP/NSAIDS, no ICIs	9,763	1,907,855	0.19 (0.18–0.19)	0.20 (0.20–0.21)		
	ICIs, no APAP/NSAIDS	39,329	631,189	2.15 (2.12–2.17)	1.80 (1.78–1.83)		
	ICIs, APAP/NSAIDS	149	4,464	1.18 (1.00–1.39)	1.01 (0.85–1.21)		
Nervous system disorders	No ICIs, no APAP/NSAIDS	3,554,577	44,038,354	Reference	Reference	0.2113	1.2529
	APAP/NSAIDS, no ICIs	106,815	1,810,803	0.72 (0.71–0.72)	0.72 (0.71–0.72)		
	ICIs, no APAP/NSAIDS	36,768	633,750	0.71 (0.70–0.71)	0.64 (0.67–0.69)		
	ICIs, APAP/NSAIDS	227	4,386	0.63 (0.55–0.72)	0.58 (0.50–0.67)		
Pregnancy, puerperium, and perinatal conditions	No ICIs, no APAP/NSAIDS	178,533	47,414,380	Reference	Reference	–	–
	APAP/NSAIDS, no ICIs	6,878	1,910,740	0.92 (0.90–0.94)	–		
	ICIs, no APAP/NSAIDS	201	670,317	0.08 (0.07–0.09)	–		
	ICIs, APAP/NSAIDS	–	–	–	–		
Product issues	No ICIs, no APAP/NSAIDS	841,327	46,751,598	Reference	Reference	0.7211	6.6357
	APAP/NSAIDS, no ICIs	11,260	1,906,358	0.33 (0.32–0.33)	0.26 (0.26–0.27)		
	ICIs, no APAP/NSAIDS	498	670,020	0.04 (0.04–0.05)	0.04 (0.04–0.05)		
	ICIs, APAP/NSAIDS	7	4,606	0.09 (0.04–0.19)	0.09 (0.04–0.21)		
Psychiatric disorders	No ICIs, no APAP/NSAIDS	2,409,604	45,183,319	Reference	Reference	0.4425	2.5594
	APAP/NSAIDS, no ICIs	80,979	1,836,639	0.80 (0.80–0.81)	0.96 (0.95–0.97)		
	ICIs, no APAP/NSAIDS	8,209	662,309	0.23 (0.23–0.24)	0.27 (0.26–0.27)		
	ICIs, APAP/NSAIDS	116	4,497	0.48 (0.40–0.58)	0.52 (0.43–0.64)		
Renal and urinary disorders	No ICIs, no APAP/NSAIDS	822,030	46,770,905	Reference	Reference	**3.5771**	**3.6312**
	APAP/NSAIDS, no ICIs	29,812	1,887,806	0.85 (0.84–0.86)	**1.07 (1.06–1.09)**		
	ICIs, no APAP/NSAIDS	20,249	650,269	1.65 (1.62–1.67)	**1.50 (1.48–1.53)**		
	ICIs, APAP/NSAIDS	431	4,182	5.07 (4.59–5.60)	**4.68 (4.19–5.24)**		
Reproductive system and breast disorders	No ICIs, no APAP/NSAIDS	354,382	47,238,540	Reference	Reference	0.8034	4.6553
	APAP/NSAIDS, no ICIs	4,769	1,912,849	0.33 (0.32–0.34)	0.37 (0.35–0.38)		
	ICIs, no APAP/NSAIDS	1,265	669,253	0.25 (0.24–0.26)	0.39 (0.36–0.41)		
	ICIs, APAP/NSAIDS	13	4,600	0.38 (0.22–0.66)	0.51 (0.28–0.92)		
Respiratory, thoracic, and mediastinal disorders	No ICIs, no APAP/NSAIDS	2,074,334	45,518,594	Reference	Reference	−0.3578	0.7770
	APAP/NSAIDS, no ICIs	71,654	1,845,964	0.81 (0.81–0.82)	0.85 (0.84–0.85)		
	ICIs, no APAP/NSAIDS	45,661	624,857	1.48 (1.46–1.49)	1.35 (1.33–1.36)		
	ICIs, APAP/NSAIDS	198	4,415	0.93 (0.81–1.07)	0.72 (0.61–0.88)		
Skin and subcutaneous tissue disorders	No ICIs, no APAP/NSAIDS	2,465,564	45,127,362	Reference	Reference	**0.6818**	**1.5996**
	APAP/NSAIDS, no ICIs	117,331	1,800,287	1.11 (1.10–1.12)	**0.99 (0.99–1.00)**		
	ICIs, no APAP/NSAIDS	37,672	632,846	1.02 (1.01–1.03)	**1.02 (1.01–1.04)**		
	ICIs, APAP/NSAIDS	461	4,152	1.81 (1.65–2.00)	**1.64 (1.47–1.83)**		
Social circumstances	No ICIs, no APAP/NSAIDS	191,608	47,401,308	Reference	Reference	−0.1756	0.8688
	APAP/NSAIDS, no ICIs	9,608	1,908,010	1.18 (1.16–1.21)	1.19 (1.16–1.22)		
	ICIs, no APAP/NSAIDS	621	669,897	0.22 (0.20–0.24)	0.19 (0.17–0.21)		
	ICIs, APAP/NSAIDS	4	4,609	0.22 (0.08–0.60)	0.18 (0.06–0.55)		
Surgical and medical procedures	No ICIs, no APAP/NSAIDS	668,826	46,924,094	Reference	Reference	0.5061	1.9905
	APAP/NSAIDS, no ICIs	10,408	1,907,210	0.38 (0.37–0.39)	0.36 (0.35–0.37)		
	ICIs, no APAP/NSAIDS	4,508	666,010	0.47 (0.45–0.48)	0.36 (0.35–0.37)		
	ICIs, APAP/NSAIDS	23	4,590	0.35 (0.23–0.53)	0.31 (0.20–0.49)		
Vascular disorders	No ICIs, no APAP/NSAIDS	898,976	46,693,954	Reference	Reference	0.3431	1.4479
	APAP/NSAIDS, no ICIs	28,752	1,888,866	0.76 (0.75–0.77)	0.85 (0.84–0.86)		
	ICIs, no APAP/NSAIDS	13,767	656,751	1.04 (1.02–1.06)	0.92 (0.90–0.94)		
	ICIs, APAP/NSAIDS	104	4,509	1.14 (0.94–1.38)	1.08 (0.88–1.33)		

Bold values indicate positive signals.

**TABLE 3:** Summary of methods used to analyze drug-drug interactions for all SOCs.

SOC	RORadj	Additive model	Multiplicative model
Blood and lymphatic system disorders	–	–	√
Cardiac disorders	–	–	–
Congenital, familial, and genetic disorders	–	√	√
Ear and labyrinth disorders	–	√	√
Endocrine disorders	–	–	√
Eye disorders	–	√	√
Gastrointestinal disorders*	√	√	–
General disorders and administration site conditions	–	–	–
Hepatobiliary disorders	–	–	–
Immune system disorders	–	–	–
Infections and infestations	–	–	–
Injury, poisoning and procedural complications	–	–	–
Investigations*	√	√	√
Metabolism and nutrition disorders	–	–	–
Musculoskeletal and connective tissue disorders	–	–	–
Neoplasms benign, malignant, and unspecified (incl cysts and polyps)	–	–	√
Nervous system disorders	–	√	√
Pregnancy, puerperium, and perinatal conditions	–	–	–
Product issues	–	√	√
Psychiatric disorders	–	√	√
Renal and urinary disorders*	√	√	√
Reproductive system and breast disorders	–	√	√
Respiratory, thoracic, and mediastinal disorders	–	–	–
Skin and subcutaneous tissue disorders*	√	√	√
Social circumstances	–	–	–
Surgical and medical procedures	–	√	√
Vascular disorders	–	√	√

* SOC, with positive signal.

### 3.3 Subset data analyses

For the four positive SOCs, we conducted a subset analysis of the co-administration of ICI with acetaminophen, non-selective COX inhibitors, and selective COX-2 inhibitors (Tables 4, 5). For gastrointestinal disorders and investigations, the co-administration of ICIs and APAP revealed positive DDI signals, which were verified using additive and multiplicative models. For renal and urinary disorders, all combinations of APAP/NSAIDs with ICIs exhibited positive DDI signals, which were confirmed via additive and multiplicative models. In skin and subcutaneous tissue disorders, the combination of NSAIDs with ICI revealed positive DDI signals, which were verified using additive and multiplicative models.

**TABLE 4:** Disproportionality analyses and drug interaction approaches for the various drug combinations.

SOC	Drug-drug interaction of interest	Exposure	Cases	Non-cases	RORcrude (95%Cl)	RORadj (95%Cl)	Additive model	Multiplicative model
Gastrointestinal disorders	ICI + APAP	No ICI, no acetaminophen	3,800,561	43,792,348	Reference	Reference	**0.1351**	**1.0660**
		ICI, no acetaminophen	69,913	603,133	1.27 (1.26–1.28)	1.30 (1.29–1.32)		
		Acetaminophen, no ICI	63,010	605,741	1.15 (1.14–1.16)	1.19 (1.18–1.20)		
		ICI, acetaminophen	263	1804	1.55 (1.36–1.76)	**1.60 (1.39–1.85)**		
	ICI + non-selective COX inhibitors	No ICI, no non-selective COX inhibitors	3,677,368	43,915,541	Reference	Reference	−0.0143	0.9465
		ICI, no non-selective COX inhibitors	69,892	602,966	1.27 (1.26–1.28)	1.32 (1.30–1.33)		
		Non-selective COX inhibitors, no ICI	158,967	1,366,403	1.27 (1.26–1.28)	1.38 (1.37–1.39)		
		ICI, non-selective COX inhibitors	284	1989	1.52 (1.34–1.72)	1.83 (1.60–2.09)		
	ICI + selective COX-2 inhibitors	No ICI, no selective COX-2 inhibitors	3,800,561	43,792,348	Reference	Reference	−0.4808	0.6570
		ICI, no selective COX-2 inhibitors	70,133	604,505	1.27 (1.26–1.28)	1.29 (1.28–1.31)		
		Selective COX-2 inhibitors, no ICI	35,774	305,739	1.28 (1.26–1.29)	1.30 (1.29–1.32)		
		ICI, selective COX-2 inhibitors	43	450	1.06 (0.78–1.45)	1.31 (0.94–1.82)		
Investigations	ICI + APAP	No ICI, no acetaminophen	2,649,591	44,943,318	Reference	Reference	**0.5167**	**1.3583**
		ICI, no acetaminophen	47,185	625,861	1.23 (1.22–1.24)	1.12 (1.11–1.13)		
		Acetaminophen, no ICI	42,623	626,128	1.12 (1.10–1.13)	1.21 (1.19–1.22)		
		ICI, acetaminophen	220	1847	1.86 (1.62–2.14)	**1.91 (1.64–2.23)**		
​	ICI + non-selective COX inhibitors	No ICI, no non-selective COX inhibitors	2,582,958	45,009,951	Reference	Reference	0.1606	1.1250
		ICI, no non-selective COX inhibitors	47,222	625,636	1.23 (1.22–1.24)	1.12 (1.11–1.13)		
		Non-selective COX inhibitors, no ICI	88,770	1,436,600	1.02 (1.01–1.03)	1.10 (1.09–1.11)		
		ICI, non-selective COX inhibitors	183	2090	1.41 (1.21–1.64)	0.96 (0.79–1.17)		
	ICI + selective COX-2 inhibitors	No ICI, no selective COX-2 inhibitors	2,649,591	44,943,318	Reference	Reference	−0.2299	0.8130
		ICI, no selective COX-2 inhibitors	47,373	627,265	1.23 (1.22–1.24)	1.19 (1.17–1.21)		
		Selective COX-2 inhibitors, no ICI	22,137	319,376	1.13 (1.12–1.15)	1.12 (1.11–1.13)		
		ICI, selective COX-2 inhibitors	32	461	1.13 (0.79–1.62)	0.90 (0.59–1.39)		
Renal and urinary disorders	ICI + APAP	No ICI, no acetaminophen	848,654	46,744,255	Reference	Reference	**2.5792**	**4.5460**
		ICI, no acetaminophen	20,537	652,509	1.66 (1.64–1.69)	1.57 (1.55–1.60)		
		Acetaminophen, no ICI	6,056	662,695	0.49 (0.48–0.51)	0.64 (0.62–0.66)		
		ICI, acetaminophen	143	1924	3.74 (3.15–4.43)	**3.43 (2.78–4.24)**		
	ICI + non-selective COX inhibitors	No ICI, no non-selective COX inhibitors	827,936	46,764,973	Reference	Reference	**4.9763**	**4.5860**
		ICI, no non-selective COX inhibitors	20,407	652,451	1.65 (1.63–1.68)	1.58 (1.55–1.60)		
		Non-selective COX inhibitors, no ICI	23,906	1,501,464	0.85 (0.84–0.87)	1.15 (1.14–1.18)		
		ICI, non-selective COX inhibitors	273	2000	6.49 (5.71–7.36)	**6.29 (5.45–7.27)**		
​	ICI + selective COX-2 inhibitors	No ICI, no selective COX-2 inhibitors	848,654	46,744,255	Reference	Reference	**7.8945**	**10.6958**
		ICI, no selective COX-2 inhibitors	20,594	654,044	1.67 (1.64–1.69)	1.58 (1.55–1.60)		
		Selective COX-2 inhibitors, no ICI	3,188	338,325	0.51 (0.49–0.53)	0.58 (0.55–0.61)		
		ICI, selective COX-2 inhibitors	86	407	9.07 (7.19–11.44)	**9.08 (6.90–11.94)**		
Skin and subcutaneous tissue disorders	ICI + APAP	No ICI, no acetaminophen	2,562,942	45,029,967	Reference	Reference	0.1744	1.1678
		ICI, no acetaminophen	37,995	635,051	1.03 (1.01–1.04)	1.08 (1.06–1.09)		
		Acetaminophen, no ICI	37,268	631,483	1.01 (1.00–1.02)	0.82 (0.81–0.83)		
		ICI, acetaminophen	138	1929	1.21 (1.02–1.44)	1.08 (0.89–1.30)		
	ICI + non-selective COX inhibitors	No ICI, no non-selective COX inhibitors	2,482,402	45,110,507	Reference	Reference	**0.8943**	**1.7280**
		ICI, no non-selective COX inhibitors	37,868	634,990	1.02 (1.01–1.03)	1.08 (1.07–1.10)		
		Non-selective COX inhibitors, no ICI	100,493	1,224,877	1.20 (1.19–1.20)	1.10 (1.09–1.11)		
		ICI, non-selective COX inhibitors	265	2008	2.11 (1.86–2.40)	**2.22 (1.90–2.59)**		
	ICI + selective COX-2 inhibitors	No ICI, no selective COX-2 inhibitors	2,562,942	45,029,967	Reference	Reference	**1.6815**	**2.5460**
		ICI, no selective COX-2 inhibitors	38,057	636,581	1.02 (1.01–1.04)	1.08 (1.07–1.09)		
		Selective COX-2 inhibitors, no ICI	19,953	321,560	1.06 (1.05–1.08)	0.94 (0.93–0.96)		
		ICI, selective COX-2 inhibitors	76	417	2.77 (2.17–3.53)	**3.65 (2.78–4.78)**		

Bold values indicate positive signals.

**TABLE 5:** Summary of methods used to analyze drug–drug interactions in four SOCs.

SOC	Exposure	RORadj	Additive model	Multiplicative model
Gastrointestinal disorders	ICI + APAP*	√	√	√
	ICI + non-selective COX inhibitors	√	–	–
	ICI + selective COX-2 inhibitors	–	–	–
Investigations	ICI + APAP*	√	√	√
	ICI + non-selective COX inhibitors	–	√	√
	ICI + selective COX-2 inhibitors	–	–	–
Renal and urinary disorders	ICI + APAP*	√	√	√
	ICI + non-selective COX inhibitors*	√	√	√
	ICI + selective COX-2 inhibitors*	√	√	√
Skin and subcutaneous tissue disorders	ICI + APAP	–	√	√
	ICI + non-selective COX inhibitors*	√	√	√
	ICI + selective COX-2 inhibitors*	√	√	√

PD-1, programmed death-1 inhibitors; PDL-1, programmed death ligand-1 inhibitors; CTLA-4, cytotoxic T-lymphocyte antigen-4, inhibitors.

* Drug combination with positive signal.

## 4 Discussion

To the best of our knowledge, this is the first pharmacovigilance study to systematically assess DDIs between ICIs and APAP/NSAIDs. Our study provides the most comprehensive real-world data regarding the clinical characteristics and DDIs associated with theco-administration of ICIs and APAP/NSAIDs. Several key findings emerged: (1) DDIs involving the co-administration of ICIs and APAP/NSAIDs occurred in four systems: gastrointestinal disorders, investigations, renal and urinary disorders, and skin and subcutaneous tissue disorders. (2) The co-administration of ICIs and APAP was associated with gastrointestinal disorders and investigations. (3) All co-administrations of APAP/NSAIDs with ICIs were associated with renal and urinary disorders. (4) The co-administrations of NSAIDs with ICIs was associated with skin and subcutaneous tissue disorders.

ICIs, such as anti-PD-1/PD-L1 and anti-CTLA-4 antibodies, act by blocking inhibitory signaling pathways, releasing the “brakes” on T cells, thereby activating adaptive immune responses and inducing anti-tumor effects. However, this process can also trigger immune overactivation and irAEs (Sharma and Allison, 2020). As the number of cancer patients receiving long-term ICI treatments increases, the interaction between these immunomodulatory agents and commonly prescribed medications become increasingly clinically relevant. Drug interactions can be categorized into pharmacodynamic and pharmacokinetic interactions. Pharmacodynamic interactions occur when different drugs affect the same physiological processes, whereas pharmacokinetic interactions involve effects on the absorption, distribution, metabolism, or excretion of a drug (Sharma and Allison, 2020). DDIs can result in either enhanced or reduced therapeutic effects, AEs, or a unique response not caused by either agent alone (Blower et al., 2005). This study focuses on DDIs that lead to increased toxicity associated with the co-administration of ICIs and APAP/NSAIDs.

NSAIDs exert their anti-inflammatory, analgesic, and antipyretic effects by inhibiting cyclooxygenase (COX) enzymes, thereby reducing the synthesis of prostaglandins (e.g., PGE_2_) (Khalil et al., 2024). COX enzymes, which are part of the myeloperoxidase family, metabolize arachidonic acid into prostaglandins and thromboxane (Pajiep et al., 2023). Prostaglandins regulate a variety of physiological processes, including tumor development, migration, differentiation, inflammation, and cell adhesion (Chandrasekharan and Simmons, 2004). COX2-mediated PGE_2_ synthesis suppresses anti-tumor immunity through multiple mechanisms, such as inhibition of myeloid cell activation, reduced recruitment of natural killer (NK) and dendritic cells (DC), induction of regulatory T-cell (Tregs), and decreased proliferation and effector function of CD4^+^/CD8^+^ T-cell (Figure 2). In animal models, COX inhibition has been shown to overcome tumor immune evasion and synergize with checkpoint blockade (Zelenay et al., 2015; Wang and Dubois, 2006).

**FIGURE 2:**
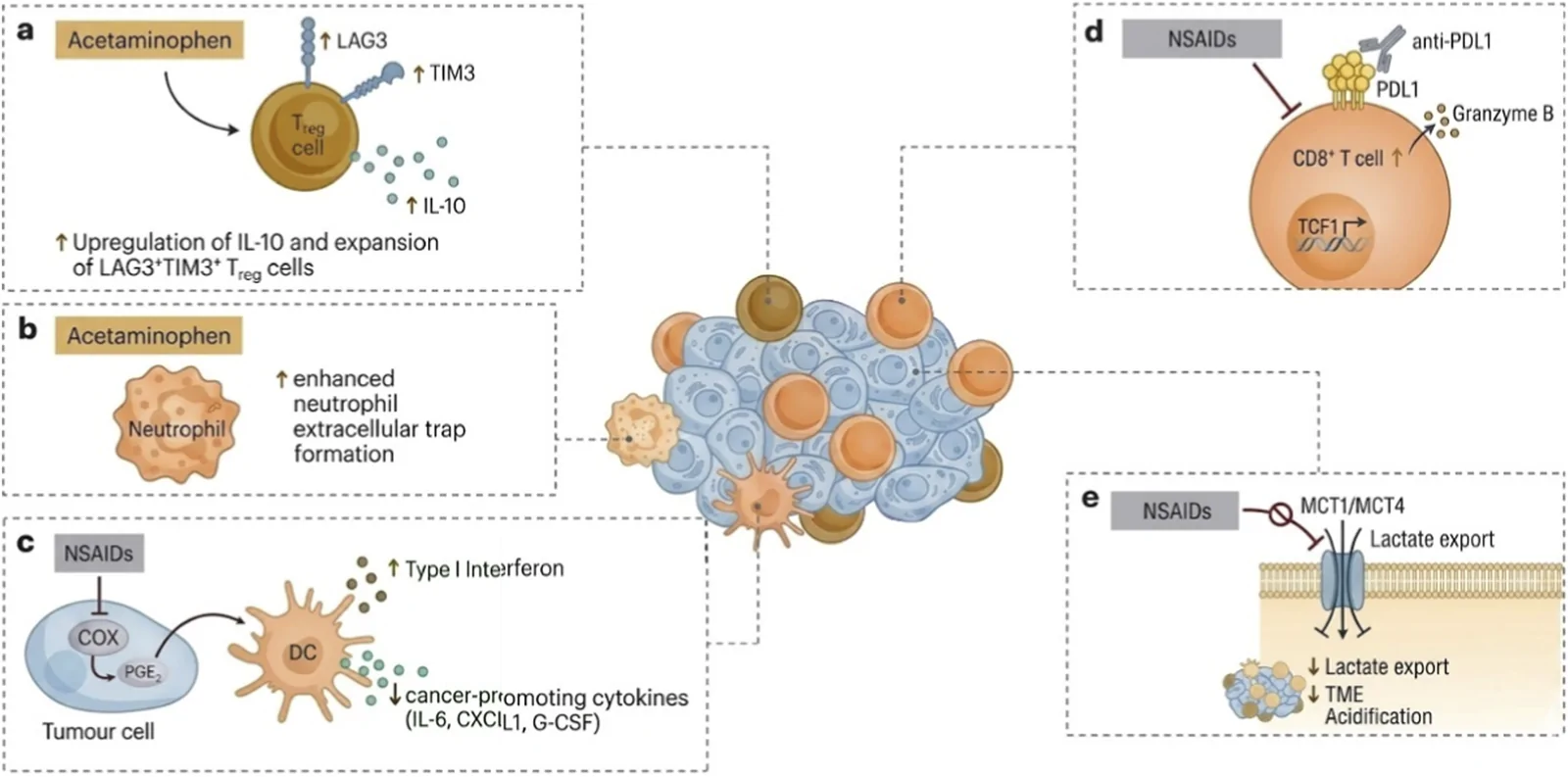
Mechanistic interactions between ICIs and APAP/NSAIDs **(a)** acetaminophen use has also been associated with the upregulation of IL-10 and the expansion of LAG3^+^TIM3^+^ regulatory T cells (Tregs) in the periphery (Stone et al., 2026) **(b)** acetaminophen use in combination with anti-PD-1 therapy is correlated with enhanced neutrophil extracellular trap (NET) formation (Stone et al., 2026) **(c)** NSAIDs inhibit cyclooxygenase (COX)-mediated prostaglandin E_2_ (PGE_2_) production, which in turn enhances type I interferon production and the secretion of cancer-promoting cytokines (interleukin-6 (IL-6), CXC-chemokine ligand 1 (CXCL1), and granulocyte colony-stimulating factor (G-CSF)) by dendritic cells (DCs) (Stone et al., 2026) **(d)** Some NSAIDs have also been shown to improve anti-PD-L1 efficacy by promoting tumor-infiltrating CD8^+^ T cells, including those expressing the T cell-specific transcription factor 1 (TCF1) or granzyme B (Stone et al., 2026) **(e)** Some NSAIDs inhibit lactate transport via monocarboxylate transporter 1 (MCT1) and MCT4, thereby improving the response to anti-PD-1 therapy by impairing tumor glycolysis and reducing acidification of the tumor microenvironment (TME) (Stone et al., 2026).

APAP, widely regarded as safe and one of the most frequently used drugs in oncology (Zhang et al., 2021), possesses analgesic and antipyretic properties similar to NSAIDs, but unlike NSAIDs, it lacks anti-inflammatory activity (Figure 2) (Zelenay et al., 2015). However, like conventional NSAIDs, it suppresses prostaglandin synthesis (Böttcher et al., 2018). The mechanism by which acetaminophen exerts its analgesic effects is not fully elucidated. Although acetaminophen lacks significant anti-inflammatory activity, it is thought to act as a selective COX-2 inhibitor (Wiffen et al., 2017). Studies have demonstrated that acetaminophen inhibits prostaglandin production in the brain and spleen, which is consistent with COX-2 inhibition (Wiffen et al., 2017). Additionally, acetaminophen may modulate the endocannabinoid system, thereby contributing to its analgesic effects (Jóźwiak-Bebenista and Nowak, 2014). Previous studies have shown that prophylactic administration of acetaminophen can limit vaccine responses in children (Hinz et al., 2008; Högestätt et al., 2005), leading the World Health Organization to recommend against its use before or during vaccination (Prymula et al., 2009).

Emerging evidence suggests that APAP may exert unintended immunomodulatory effects, raising concerns about its potential to compromise anti-tumor immune responses (Falup-Pecurariu et al., 2017). Mechanistic studies have demonstrated that APAP can inhibit T-cell proliferation, suppress interferon-gamma (IFN-γ) production, and promote regulatory T-cell (Treg) expansion—actions that collectively impair cytotoxic immunity (Falup-Pecurariu et al., 2017). A meta-analysis identified a consistent association between APAP administration and worsened clinical outcomes in cancer patients receiving ICIs therapy, with nearly a 30% increased risk of death or disease progression in APAP-exposed individuals (ACIP Vaccine Administration Guidelines for Immunization, 2022).

Studies have also explored the impact of NSAID use on ICI treatment outcomes, yielding mixed results -ranging from improved efficacy and survival in specific clinical contexts to neutral or even detrimental effects (Falup-Pecurariu et al., 2017; Bessede et al., 2022; Lin et al., 2025; Wang et al., 2020). Two meta-analyses confirmed that the concurrent administration of NSAIDs was not significantly associated with prognosis in patients receiving ICI therapy (Zhang et al., 2021; Mao et al., 2022). However, these studies failed to distinguish between APAP and NSAIDs, despite the fact that both are often erroneously sclassified as NSAIDs. From a clinical perspective, there is little practical difference between the two, yet from an immunotherapy standpoint, this failure to differentiate could introduce significant bias.

ICIs stimulate the immune system, thereby enhancing anti-tumor responses (Cortellini et al., 2020). While effective in combating malignancy, this immune stimulation can trigger immune-mediated inflammatory responses, known as irAEs, which can occur in any organ system of the body (Bessede et al., 2022). Some researchers propose that activated effector T-cell attack normal, non-tumor tissues while exerting their anti-tumor activity (Kanai et al., 2021; Wei et al., 2018). T-cell receptor (TCR) sequencing studies have provided supportfor this theory (Pauken et al., 2019; Khan and Gerber, 2020). In patients treated with Ipilimumab, researchers observed greater diversity of CD4^+^ and CD8^+^ T-cell in those with irAE compared to those without significant adverse reactions (Ronen et al., 2022), offering mechanistic insight into organ-specific toxicities.

ICIs can induce a range of kidney-related irAEs, including AKI, which often presents as acute interstitial nephritis (AIN), though glomerular disease and electrolyte disturbances have also been reported (Porciello et al., 2022). The incidence of AKI in patients receiving ICI therapy can be as high as 17%, but ICI-induced AKI is estimated to account for 3%–5% of these cases (Porciello et al., 2022). Risk factors for ICI-AKI include baseline chronic kidney disease (CKD), combination treatment with anti-CTLA-4 and anti-PD-1/PD-L1 agents, prior or concurrent extrarenal irAEs, and the use of drugs associated with AIN (Sanromán et al., 2023; Oh et al., 2017; Herrmann et al., 2025). NSAIDs can bind to renal tubules and trigger immune responses that are normally suppressed, thereby resulting in kidney damage. This synergistic effect, when combined with ICIs, can lead to AKI (Gupta et al., 2021). A meta-analysis identified NSAID administration as a risk factor for AKI in patients receiving ICI (Zhang et al., 2021). A recent study using the WHO’s Vigibase database found that NSAIDs were associated with increased reports of nephritis in the context of ICI therapy, suggesting the need for careful consideration in clinical prescription (Laurent et al., 2025). Our study further confirms that renal toxicity arises from synergistic effects and reveals for the first time the correlation between the co-administration of NSAIDs and ICIs and toxicity in the gastrointestinal, skin, and subcutaneous tissue systems.

Cutaneous irAEs are a common consequence of ICI treatment, as immune system overactivation leadsto dermatologic toxicities (Seethapathy et al., 2019). These cutaneous irAEs are among the most frequent complications of ICI therapy (Cortazar et al., 2020; Perazella and Shirali, 2018). Many present similarly to primary dermatoses and may share features with autoimmune skin disorders (Cortazar et al., 2020; Laurent et al., 2025; Tattersall et al., 2020). Cutaneous irAEs occur in up to 30%–50% of patients receiving ICIs therapy (Perazella and Shirali, 2018; Laurent et al., 2025), though most are mild and can be managed without interrupting immunotherapy. Interestingly, these reactions can mimic a wide range of primary dermatoses, suggesting a T-cell-mediated mechanism common to various skin disorders.

Gastrointestinal irAEs are the second most common following cutaneous involvement and are often the primary reason for discontinuation of immunotherapy (Haanen et al., 2018; Curry et al., 2017; Villadolid and Amin, 2015). Diarrhea is the most frequently reported symptom, often accompanied by abdominal pain, bloating, rectal bleeding, or fever (Lacouture and Sibaud, 2018; Sibaud, 2018). APAP-induced gastrointestinal toxicity is a significant aspect of its safety profile, with reported rates of gastrointestinal AEs ranging from 5.3% to 9.0% (Schneider et al., 2021). While less severe than NSAID-related gastrointestinal damage, APAP can cause dyspepsia, nausea, abdominal pain, and in rare cases, gastric erosions, ulcers, and bleeding (García Rodríguez and Hernández-Díaz, 2001). Risk factors for more severe outcomes include age, a history of peptic ulcers, alcohol consumption, and prolonged fasting (Prescott, 2000).

Our study has certain limitations. First, the FAERS database is a spontaneous reporting system (SRS) with inherent limitations, including underreporting and inconsistent reporting quality. It was not feasible to quantify adverse reaction signals based on the total number of AEs using the collected data; instead, the study aimed to provide a qualitative indicator solely based on the signal intensity between the drugs and the corresponding reactions. Second, compared with clinical trials and cohort studies, SRS data are generally less reliable due to incomplete clinical details, residual confounding factors, and missing denominator data. The identification and reporting of AEs in SRS are subject to less stringent quality control. Third, it is challenging to identify significant risk factors between disorders and drugs due to deficiencies in the documentation of preexisting disorders and comorbidities, which may influence the assessment of disease causality. Furthermore, this study was not restricted to a specific disease, which differs significantly from clinical trials. Fourth, this study used SOCs as the target reactions to encompass all AEs; however, the scope was overly broad, and subgroup analyses were not performed. Fifth, the medications listed in the same report are not entirely equivalent to those actually taken by the patient concurrently, and there may be reporting bias associated with combined medication use. For DDIs, the relative reporting rate of drug-induced AEs may be overestimated if there is a lack of information on either of the two drugs involved (Riveiro-Barciela et al., 2024; Moore and Scheiman, 2018; Cheung and Brain, 2020; Noguchi et al., 2021; Sakaeda et al., 2013). Sixth, calculations, justifications, and power analyses for the selected sample size were not conducted, as the study intended to include all eligible ADRs. Seventh, the FAERS database has inherent reporting biases, including the Weber effect (reporting rates increase in the first 2 years after a drug’s launch and then decline), the ripple effect (reporting of other drugs in the same class is expedited due to the notoriety of one drug), and the masking or cloaking effect (reports of the same adverse event linked to other drugs can suppress signal scores). Finally, data mining revealed imperfect reporting, with inaccuracies and incomplete entries (e.g., drug dosage and duration of use), which may introduce analytical bias. Nevertheless, our findings, despite being limited by the inherent constraints of the FAERS database, shed light on the DDIs between ICIs and APAP/NSAIDs and provide a framework for more rigorous follow-up research to validate these findings.

## 5 Conclusion

Our study identified four types of DDIs associated with the co-administration of ICIs and APAP/NSAIDs using real-world data from the FAERS. Therefore, close attention should be paid when these two classes of drugs are co-administered. Preclinical trials are required to explore the underlying mechanisms of these interactions, and further well-designed, robust clinical studies are necessary to clarify these relationships, elucidate the risk of DDIs associated with this combination therapy, and provide more granular details.

## Data Availability

The datasets presented in this study can be found in online repositories. The names of the repository/repositories and accession number(s) can be found in the article/Supplementary Material.

## References

[B1] ACIP Vaccine Administration Guidelines for Immunization (2022). Atlanta, GA: Centers for Disease Control and Prevention (CDC). Available online at: https://www.cdc.gov/vaccines/hcp/acip-recs/generalrecs/administration.html (Accessed March 6, 2026).

[B2] Bessede A. Marabelle A. Guégan J. P. Danlos F. X. Cousin S. Peyraud F. (2022). Impact of acetaminophen on the efficacy of immunotherapy in cancer patients. Ann. Oncol. 33 (9), 909–915. 10.1016/j.annonc.2022.05.010 35654248

[B3] Blower P. de Wit R. Goodin S. Aapro M. (2005). Drug-drug interactions in oncology: why are they important and can they be minimized? Crit. Rev. Oncol. Hematol. 55 (2), 117–142. 10.1016/j.critrevonc.2005.03.007 15890526

[B4] Bonavita E. Bromley C. P. Jonsson G. Pelly V. S. Sahoo S. Walwyn-Brown K. (2020). Antagonistic inflammatory phenotypes dictate tumor fate and response to immune checkpoint blockade. Immunity 53 (6), 1215–1229.e8. 10.1016/j.immuni.2020.10.020 33220234 PMC7772804

[B5] Böttcher J. P. Bonavita E. Chakravarty P. Blees H. Cabeza-Cabrerizo M. Sammicheli S. (2018). NK cells stimulate recruitment of cDC1 into the tumor microenvironment promoting cancer immune control. Cell 172 (5), 1022–1037.e14. 10.1016/j.cell.2018.01.004 29429633 PMC5847168

[B6] Capogrosso Sansone A. Convertino I. Galiulo M. T. Salvadori S. Pieroni S. Knezevic T. (2017). Muscular adverse drug reactions associated with proton pump inhibitors: a disproportionality analysis using the Italian national network of pharmacovigilance database. Drug Saf. 40 (10), 895–909. 10.1007/s40264-017-0564-8 28681266

[B7] Chandrasekharan N. V. Simmons D. L. (2004). The cyclooxygenases. Genome Biol. 5 (9), 241. 10.1186/gb-2004-5-9-241 15345041 PMC522864

[B8] Cheung V. T. F. Brain O. (2020). Immunotherapy induced enterocolitis and gastritis - what to do and when? Best. Pract. Res. Clin. Gastroenterol. 48-49, 101703. 10.1016/j.bpg.2020.101703 33317787

[B9] Cortazar F. B. Kibbelaar Z. A. Glezerman I. G. Abudayyeh A. Mamlouk O. Motwani S. S. (2020). Clinical features and outcomes of immune checkpoint inhibitor-associated AKI: a multicenter study. J. Am. Soc. Nephrol. 31 (2), 435–446. 10.1681/ASN.2019070676 31896554 PMC7003302

[B10] Cortellini A. Tucci M. Adamo V. Stucci L. S. Russo A. Tanda E. T. (2020). Integrated analysis of concomitant medications and oncological outcomes from PD-1/PD-L1 checkpoint inhibitors in clinical practice. J. Immunother. Cancer 8 (2), e001361. 10.1136/jitc-2020-001361 33154150 PMC7646355

[B11] Curry J. L. Tetzlaff M. T. Nagarajan P. Drucker C. Diab A. Hymes S. R. (2017). Diverse types of dermatologic toxicities from immune checkpoint blockade therapy. J. Cutan. Pathol. 44 (2), 158–176. 10.1111/cup.12858 27859479

[B12] Falup-Pecurariu O. Man S. C. Neamtu M. L. Chicin G. Baciu G. Pitic C. (2017). Effects of prophylactic ibuprofen and paracetamol administration on the immunogenicity and reactogenicity of the 10-valent pneumococcal non-typeable Haemophilus influenzae protein D conjugated vaccine (PHiD-CV) co-administered with DTPa-combined vaccines in children: an open-label, randomized, controlled, non-inferiority trial. Hum. Vaccin Immunother. 13 (3), 649–660. 10.1080/21645515.2016.1223001 27541270 PMC5360152

[B13] García Rodríguez L. A. Hernández-Díaz S. (2001). Relative risk of upper gastrointestinal complications among users of acetaminophen and nonsteroidal anti-inflammatory drugs. Epidemiology 12 (5), 570–576. 10.1097/00001648-200109000-00018 11505178

[B14] Gupta S. Short S. A. P. Sise M. E. Prosek J. M. Madhavan S. M. Soler M. J. (2021). Acute kidney injury in patients treated with immune checkpoint inhibitors. J. Immunother. Cancer 9 (10), e003467. 10.1136/jitc-2021-003467 34625513 PMC8496384

[B15] Haanen JBAG Carbonnel F. Robert C. Kerr K. M. Peters S. Larkin J. (2018). ESMO guidelines committee. Management of toxicities from immunotherapy: ESMO clinical practice guidelines for diagnosis, treatment and follow-up. Ann. Oncol. 29 (4), iv264–iv266. 10.1093/annonc/mdy162 29917046

[B16] Herrmann S. M. Abudayyeh A. Gupta S. Gudsoorkar P. Klomjit N. Motwani S. S. (2025). Diagnosis and management of immune checkpoint inhibitor-associated nephrotoxicity: a position statement from the American society of Onco-nephrology. Kidney Int. 107 (1), 21–32. 10.1016/j.kint.2024.09.017 39455026

[B17] Hinz B. Cheremina O. Brune K. (2008). Acetaminophen (Paracetamol) is a selective cyclooxygenase-2 inhibitor in man. FASEB J. 22 (2), 383–390. 10.1096/fj.07-8506com 17884974

[B18] Högestätt E. D. Jönsson B. A. Ermund A. Andersson D. A. Björk H. Alexander J. P. (2005). Conversion of acetaminophen to the bioactive N-acylphenolamine AM404 via fatty acid amide hydrolase-dependent arachidonic acid conjugation in the nervous system. J. Biol. Chem. 280 (36), 31405–31412. 10.1074/jbc.M501489200 15987694

[B19] Jóźwiak-Bebenista M. Nowak J. Z. (2014). Paracetamol: mechanism of action, applications and safety concern. Acta Pol. Pharm. 71 (1), 11–23. 24779190

[B20] Kanai O. Ito T. Saito Z. Yamamoto Y. Fujita K. Okamura M. (2021). Effect of cyclooxygenase inhibitor use on immunotherapy efficacy in non-small cell lung cancer. Thorac. Cancer 12 (6), 949–957. 10.1111/1759-7714.13845 33559253 PMC7952791

[B21] Khalil N. A. Ahmed E. M. Tharwat T. Mahmoud Z. (2024). NSAIDs between past and present; a long journey towards an ideal COX-2 inhibitor lead. RSC Adv. 14 (42), 30647–30661. 10.1039/d4ra04686b 39324041 PMC11423417

[B22] Khan S. Gerber D. E. (2020). Autoimmunity, checkpoint inhibitor therapy and immune-related adverse events: a review. Semin. Cancer Biol. 64, 93–101. 10.1016/j.semcancer.2019.06.012 31330185 PMC6980444

[B23] Lacouture M. Sibaud V. (2018). Toxic side effects of targeted therapies and immunotherapies affecting the skin, oral mucosa, hair, and nails. Am. J. Clin. Dermatol 19 (Suppl. 1), 31–39. 10.1007/s40257-018-0384-3 30374901 PMC6244569

[B24] Laurent L. Abbar B. Bihan K. Dumas E. Jochum F. Lebrun-Vignes B. (2025). Comedications associated with immune-related adverse events from immune-checkpoint inhibitors. Clin. Pharmacol. Ther. 118 (3), 593–599. 10.1002/cpt.3721 40521636 PMC12355013

[B25] Li Y. Jia X. Liu M. Zhu Z. Qiao H. Jiang J. (2026). Impact of NSAID type, initiation timing, duration and dose on clinical outcomes of immunotherapy in NSCLC: a multicenter two-cohort study. J. Immunother. Cancer 14 (4), e014360. 10.1136/jitc-2025-014360 41922087 PMC13052584

[B26] Lin Y. Liao Y. Shen J. (2025). Acetaminophen use and prognosis in cancer patients treated with immune checkpoint inhibitors: evidence from a meta-analysis. Front. Immunol. 16, 1682686. 10.3389/fimmu.2025.1682686 41357190 PMC12678302

[B27] Liu C. Wei W. Yang L. Li J. Yi C. Pu Y. (2023). Incidence and risk factors of acute kidney injury in cancer patients treated with immune checkpoint inhibitors: a systematic review and meta-analysis. Front. Immunol. 14, 1173952. 10.3389/fimmu.2023.1173952 37313406 PMC10258324

[B28] Maintenance and support service organization. Support documentation: MedDRA version 23. Available online at: https://www.meddra.org/how-to-use/support-documentation/English (Accessed October 6, 2021).

[B29] Mao Z. Jia X. Jiang P. Wang Q. Zhang Y. Li Y. (2022). Effect of concomitant use of analgesics on prognosis in patients treated with immune checkpoint inhibitors: a systematic review and meta-analysis. Front. Immunol. 13, 861723. 10.3389/fimmu.2022.861723 35603146 PMC9120587

[B30] Michielin O. Lalani A. K. Robert C. Sharma P. Peters S. (2022). Defining unique clinical hallmarks for immune checkpoint inhibitor-based therapies. J. Immunother. Cancer 10 (1), e003024. 10.1136/jitc-2021-003024 35078922 PMC8796265

[B31] Moore N. Scheiman J. M. (2018). Gastrointestinal safety and tolerability of oral non-aspirin over-the-counter analgesics. Postgrad. Med. 130 (2), 188–199. 10.1080/00325481.2018.1429793 29417856

[B32] Noguchi Y. Tachi T. Teramachi H. (2019). Review of statistical methodologies for detecting drug-drug interactions using spontaneous reporting systems. Front. Pharmacol. 10, 1319. 10.3389/fphar.2019.01319 31780939 PMC6857477

[B33] Noguchi Y. Tachi T. Teramachi H. (2020). Comparison of signal detection algorithms based on frequency statistical model for drug-drug interaction using spontaneous reporting systems. Pharm. Res. 37 (5), 86. 10.1007/s11095-020-02801-3 32356247

[B34] Noguchi Y. Tachi T. Teramachi H. (2021). Detection algorithms and attentive points of safety signal using spontaneous reporting systems as a clinical data source. Brief. Bioinform 22 (6), bbab347. 10.1093/bib/bbab347 34453158

[B35] Oh D. Y. Cham J. Zhang L. Fong G. Kwek S. S. Klinger M. (2017). Immune toxicities elicted by CTLA-4 blockade in cancer patients are associated with early diversification of the T-cell repertoire. Cancer Res. 77 (6), 1322–1330. 10.1158/0008-5472.CAN-16-2324 28031229 PMC5398199

[B36] Pajiep M. Lapeyre-Mestre M. Despas F. (2023). Drug-drug interactions of protein kinase inhibitors in chronic myeloid leukaemia patients: a study using the French health insurance database. Fundam. Clin. Pharmacol. 37 (5), 994–1005. 10.1111/fcp.12899 37069127

[B37] Pauken K. E. Dougan M. Rose N. R. Lichtman A. H. Sharpe A. H. (2019). Adverse events following cancer immunotherapy: obstacles and opportunities. Trends Immunol. 40 (6), 511–523. 10.1016/j.it.2019.04.002 31053497 PMC6527345

[B38] Perazella M. A. Shirali A. C. (2018). Nephrotoxicity of cancer immunotherapies: past, present and future. J. Am. Soc. Nephrol. 29 (8), 2039–2052. 10.1681/ASN.2018050488 29959196 PMC6065079

[B39] Porciello N. Franzese O. D'Ambrosio L. Palermo B. Nisticò P. (2022). T-cell repertoire diversity: friend or foe for protective antitumor response? J. Exp. Clin. Cancer Res. 41 (1), 356. 10.1186/s13046-022-02566-0 36550555 PMC9773533

[B40] Prescott L. F. (2000). Paracetamol, alcohol and the liver. Br. J. Clin. Pharmacol. 49 (4), 291–301. 10.1046/j.1365-2125.2000.00167.x 10759684 PMC2014937

[B41] Prymula R. Siegrist C. A. Chlibek R. Zemlickova H. Vackova M. Smetana J. (2009). Effect of prophylactic paracetamol administration at time of vaccination on febrile reactions and antibody responses in children: two open-label, randomised controlled trials. Lancet 374 (9698), 1339–1350. 10.1016/S0140-6736(09)61208-3 19837254

[B42] Ribas A. Wolchok J. D. (2018). Cancer immunotherapy using checkpoint blockade. Science 359 (6382), 1350–1355. 10.1126/science.aar4060 29567705 PMC7391259

[B43] Riveiro-Barciela M. Carballal S. Díaz-González Á. Mañosa M. Gallego-Plazas J. Cubiella J. (2024). Management of liver and gastrointestinal toxicity induced by immune checkpoint inhibitors: position statement of the AEEH-AEG-SEPD-SEOM-GETECCU. Gastroenterol. Hepatol. 47 (4), 401–432. 10.1016/j.gastrohep.2023.10.009 38228461

[B44] Ronen D. Bsoul A. Lotem M. Abedat S. Yarkoni M. Amir O. (2022). Exploring the mechanisms underlying the cardiotoxic effects of immune checkpoint inhibitor therapies. Vaccines (Basel) 10 (4), 540. 10.3390/vaccines10040540 35455289 PMC9031363

[B45] Sakaeda T. Tamon A. Kadoyama K. Okuno Y. (2013). Data mining of the public version of the FDA adverse event reporting system. Int. J. Med. Sci. 10 (7), 796–803. 10.7150/ijms.6048 23794943 PMC3689877

[B46] Sanromán Á. F. Joshi K. Au L. Chain B. Turajlic S. (2023). TCR sequencing: applications in immuno-oncology research. Immunooncol Technol. 17, 100373. 10.1016/j.iotech.2023.100373 36908996 PMC9996383

[B47] Schneider B. J. Naidoo J. Santomasso B. D. Lacchetti C. Adkins S. Anadkat M. (2021). Management of immune-related adverse events in patients treated with immune checkpoint inhibitor therapy: ASCO guideline update. J. Clin. Oncol. 39 (36), 4073–4126. 10.1200/JCO.21.01440 34724392

[B48] Seethapathy H. Zhao S. Chute D. F. Zubiri L. Oppong Y. Strohbehn I. (2019). The incidence, causes, and risk factors of acute kidney injury in patients receiving immune checkpoint inhibitors. Clin. J. Am. Soc. Nephrol. 14 (12), 1692–1700. 10.2215/CJN.00990119 31672794 PMC6895474

[B49] Sharma P. Allison J. P. (2020). Dissecting the mechanisms of immune checkpoint therapy. Nat. Rev. Immunol. 20 (2), 75–76. 10.1038/s41577-020-0275-8 31925406

[B50] Sibaud V. (2018). Dermatologic reactions to immune checkpoint inhibitors: skin toxicities and immunotherapy. Am. J. Clin. Dermatol 19 (3), 345–361. 10.1007/s40257-017-0336-3 29256113

[B51] Sohi G. Lao N. Caraceni A. Moulin D. E. Zimmermann C. Herx L. (2021). Nonopioid drug combinations for cancer pain: a systematic review. Pain Rep. 7 (2), e995. 10.1097/PR9.0000000000000995 35261931 PMC8893303

[B52] Stone S. McPherson J. P. Kulkarni R. P. Young A. (2026). The impact of concomitant medications on treatment outcomes in patients with cancer receiving immune checkpoint inhibitors. Nat. Rev. Cancer 26 (2), 137–158. 10.1038/s41568-025-00890-z 41339541 PMC13633680

[B53] Tattersall I. W. Leventhal J. S. (2020). Cutaneous toxicities of immune checkpoint inhibitors: the role of the dermatologist. Yale J. Biol. Med. 93 (1), 123–132. 32226342 PMC7087048

[B54] Thakrar B. T. Grundschober S. B. Doessegger L. (2007). Detecting signals of drug-drug interactions in a spontaneous reports database. Br. J. Clin. Pharmacol. 64 (4), 489–495. 10.1111/j.1365-2125.2007.02900.x 17506784 PMC2048563

[B55] U.S. Food and Drug Administration (2021). FDA adverse event reporting system (FAERS) quarterly data extract files. Available online at: https://fis.fda.gov/extensions/fpd-qde-faers/fpd-qde-faers.html (Accessed October 20, 2021).

[B56] van Puijenbroek E. P. Egberts A. C. Heerdink E. R. Leufkens H. G. (2000). Detecting drug-drug interactions using a database for spontaneous adverse drug reactions: an example with diuretics and non-steroidal anti-inflammatory drugs. Eur. J. Clin. Pharmacol. 56 (9-10), 733–738. 10.1007/s002280000215 11214785

[B57] van Puijenbroek E. P. Bate A. Leufkens H. G. Lindquist M. Orre R. Egberts A. C. (2002). A comparison of measures of disproportionality for signal detection in spontaneous reporting systems for adverse drug reactions. Pharmacoepidemiol Drug Saf. 11 (1), 3–10. 10.1002/pds.668 11998548

[B58] Villadolid J. Amin A. (2015). Immune checkpoint inhibitors in clinical practice: update on management of immune-related toxicities. Transl. Lung Cancer Res. 4 (5), 560–575. 10.3978/j.issn.2218-6751.2015.06.06 26629425 PMC4630514

[B59] Wang D. Dubois R. N. (2006). Prostaglandins and cancer. Gut. 55 (1), 115–122. 10.1136/gut.2004.047100 16118353 PMC1856377

[B60] Wang S. J. Khullar K. Kim S. Yegya-Raman N. Malhotra J. Groisberg R. (2020). Effect of cyclo-oxygenase inhibitor use during checkpoint blockade immunotherapy in patients with metastatic melanoma and non-small cell lung cancer. J. Immunother. Cancer 8 (2), e000889. 10.1136/jitc-2020-000889 33020239 PMC7537331

[B61] Wei S. C. Duffy C. R. Allison J. P. (2018). Fundamental mechanisms of immune checkpoint blockade therapy. Cancer Discov. 8 (9), 1069–1086. 10.1158/2159-8290.CD-18-0367 30115704

[B62] Wiffen P. J. Derry S. Moore R. A. McNicol E. D. Bell R. F. Carr D. B. (2017). Oral paracetamol (acetaminophen) for cancer pain. Cochrane Database Syst. Rev. 7(7), CD012637. 10.1002/14651858.CD012637.pub2 28700092 PMC6369932

[B63] Yang H. Liu Z. Li R. Huang R. Peng X. (2023). The association between aspirin use and immune-related adverse events in specific cancer patients receiving ICIs therapy: analysis of the FAERS database. Front. Pharmacol. 14, 1259628. 10.3389/fphar.2023.1259628 38035011 PMC10686414

[B64] Yue Z. Shi J. Jiang P. Sun H. (2014). Acute kidney injury during concomitant use of valacyclovir and loxoprofen: detecting drug-drug interactions in a spontaneous reporting system. Pharmacoepidemiol Drug Saf. 23 (11), 1154–1159. 10.1002/pds.3626 24788910

[B65] Zelenay S. van der Veen A. G. Böttcher J. P. Snelgrove K. J. Rogers N. Acton S. E. (2015). Cyclooxygenase-dependent tumor growth through evasion of immunity. Cell 162 (6), 1257–1270. 10.1016/j.cell.2015.08.015 26343581 PMC4597191

[B66] Zhang Y. Chen H. Chen S. Li Z. Chen J. Li W. (2021). The effect of concomitant use of statins, NSAIDs, low-dose aspirin, metformin and beta-blockers on outcomes in patients receiving immune checkpoint inhibitors: a systematic review and meta-analysis. Oncoimmunology 10 (1), 1957605. 10.1080/2162402X.2021.1957605 34377596 PMC8331004

