# Supplementary material for: Adverse reactions of immune checkpoint inhibitors combined with acetaminophen/non-steroidal anti-inflammatory drugs (NSAIDs): a pharmacovigilance analysis of drug–drug interactions
Source: Front Pharmacol. 2026 Oct 9;17:1833275. doi: 10.3389/fphar.2026.1833275 (PMC13690646; doi:10.3389/fphar.2026.1833275)
Supplement: Supplementary file 1 [file Table1.docx]

**Table S1. 27 Standardized MedDRA Query “System Organ Class”**

| **SOC** | **Code** |
| --- | --- |
| Blood and lymphatic system disorders | 10005329 |
| Cardiac disorders | 10007541 |
| Congenital, familial and genetic disorders | 10010331 |
| Ear and labyrinth disorders | 10013993 |
| Endocrine disorders | 10014698 |
| Eye disorders | 10015919 |
| Gastrointestinal disorders | 10017947 |
| General disorders and administration site conditions | 10018065 |
| Hepatobiliary disorders | 10019805 |
| Immune system disorders | 10021428 |
| Infections and infestations | 10021881 |
| Injury, poisoning and procedural complications | 10022117 |
| Investigations | 10022891 |
| Metabolism and nutrition disorders | 10027433 |
| Musculoskeletal and connective tissue disorders | 10028395 |
| Neoplasms benign, malignant and unspecified (incl cysts and polyps) | 10029104 |
| Nervous system disorders | 10029205 |
| Pregnancy, puerperium and perinatal conditions | 10036585 |
| Product issues | 10077536 |
| Psychiatric disorders | 10037175 |
| Renal and urinary disorders | 10038359 |
| Reproductive system and breast disorders | 10038604 |
| Respiratory, thoracic and mediastinal disorders | 10038738 |
| Skin and subcutaneous tissue disorders | 10040785 |
| Social circumstances | 10041244 |
| Surgical and medical procedures | 10042613 |
| Vascular disorders | 10047065 |

SOC, Standardized MedDRA Query “System Organ Class”

**Table S2. Acetaminophen and non-steroidal anti-inflammatory drugs (NSAIDs) included in this study**

| Classification | Medications |
| --- | --- |
| Acetaminophen | Acetaminophen |
| Non-selective COX inhibitors | Balsalazide, Bromfenac, Carprofen, Diclofenac, Diflunisal, Etodolac, Fenoprofen, Flurbiprofen, Ibuprofen, Indomethacin, Ketoprofen, Ketorolac, Mefenamic, Meloxicam, Nabumetone, Naproxen, Nepafenac, Oxaprozin, Oxyphenbutazone, Phenylbutazone, Piroxicam, Salicylic, Sulfasalazine, Sulindac, Suprofen, Tolmetin, Loxoprofen |
| Selective COX-2 inhibitors | Valdecoxib, Celecoxib, Rofecoxib, Parecoxib, Etoricoxib |
